# Biosynthesis of Peptide Hydrogel–Titania Nanoparticle Composites with Antibacterial Properties

**DOI:** 10.3390/gels9120940

**Published:** 2023-11-30

**Authors:** Roya Binaymotlagh, Farid Hajareh Haghighi, Enea Gino Di Domenico, Francesca Sivori, Mauro Truglio, Alessandra Del Giudice, Ilaria Fratoddi, Laura Chronopoulou, Cleofe Palocci

**Affiliations:** 1 Department of Chemistry, Sapienza University of Rome, Piazzale Aldo Moro 5, 00185 Rome, Italy; roya.binaymotlagh@uniroma1.it (R.B.); farid.hajarehhaghighi@uniroma1.it (F.H.H.); alessandra.delgiudice@uniroma1.it (A.D.G.); ilaria.fratoddi@uniroma1.it (I.F.); cleofe.palocci@uniroma1.it (C.P.); 2 Department of Biology and Biotechnology “C. Darwin”, Sapienza University of Rome, Piazzale Aldo Moro 5, 00185 Rome, Italy; enea.didomenico@uniroma1.it; 3 Microbiology and Virology, San Gallicano Dermatological Institute, IRCCS, 00144 Rome, Italy; francesca.sivori@ifo.it (F.S.); mauro.truglio@ifo.it (M.T.); 4 Research Center for Applied Sciences to the Safeguard of Environment and Cultural Heritage (CIABC), Sapienza University of Rome, Piazzale Aldo Moro 5, 00185 Rome, Italy

**Keywords:** peptide-based hydrogels, titania nanoparticles, antibacterial properties, hydrogel composites, *Staphylococcus aureus*, MRSA, skin

## Abstract

The photoantibacterial properties of titania nanoparticles (TiO_2_NPs) are attracting much interest, but the separation of their suspension limits their application. In this study, the encapsulation of commercial TiO_2_NPs within self-assembling tripeptide hydrogels to form hgel-TiO_2_NP composites with significant photoantibacterial properties is reported. The Fmoc-Phe_3_ hydrogelator was synthesized via an enzymatic method. The resulting composite was characterized with DLS, ζ-potential, SAXS, FESEM-EDS and rheological measurements. Two different concentrations of TiO_2_NPs were used. The results showed that, by increasing the TiO_2_NP quantity from 5 to 10 mg, the value of the elastic modulus doubled, while the swelling ratio decreased from 63.6 to 45.5%. The antimicrobial efficacy of hgel-TiO_2_NPs was tested against a laboratory *Staphylococcus aureus* (*S. aureus*) strain and two methicillin-resistant *S. aureus* (MRSA) clinical isolates. Results highlighted a concentration-dependent superior antibacterial activity of hgel-TiO_2_NPs over TiO_2_NPs in the dark and after UV photoactivation. Notably, UV light exposure substantially increased the biocidal action of hgel-TiO_2_NPs compared to TiO_2_NPs. Surprisingly, in the absence of UV light, both composites significantly increased *S. aureus* growth relative to control groups. These findings support the role of hgel-TiO_2_NPs as promising biocidal agents in clinical and sanitation contexts. However, they also signal concerns about TiO_2_NP exposure influencing *S. aureus* virulence.

## 1. Introduction

In recent decades, research focusing on nano-sized drugs with specific physical and chemical properties has opened new ways for developing innovative pharmaceutical products, especially with antimicrobial activities. Due to paramount features such as unique optical, electrical, chemical and mechanical features and high surface-to-volume ratios, nanoparticles are attracting much attention in this field [1,2,3]. These significant properties have placed them at the center of attention for the development of novel diagnostic and therapeutic tools [4]. Among different types of nanoparticles (NPs), metal oxide NPs have shown significant antimicrobial activities and are considered good candidates as bactericidal agents [5,6,7,8,9,10]. 

As antibacterial agents, titania nanoparticles (TiO_2_NPs) are attracting growing interest due to their chemical stability, biocompatibility and proven antibacterial properties [11,12,13]. Thanks to their low toxicity, TiO_2_NPs are used in many applications, such as cosmetic formulations, as an ingredient in sunscreens, eyeshadows and powders. However, it has been shown that both the crystalline structure and size of TiO_2_NPs (anatase) have an effect on their safe use [14,15]. The applications of TiO_2_NPs include the indoor disinfection of walls and floor tiles in hospitals and public spaces and their use as insecticides for sterilization [16]. Another application relates to air purification for infection prevention [17,18]. It has been proved that TiO_2_NPs show effective antibacterial activity against both Gram-negative and Gram-positive bacteria [19]. It is well known that, after exposure to light, TiO_2_NPs, as semi-conductor NPs, produce an electron–hole that reacts with oxygen species and is highly reactive upon facing cell membranes and cytoplasmic materials [20]. Production of reactive oxygen species (ROS) is the main mechanism responsible for the photoantibacterial properties of TiO_2_NPs, which may degrade the integrity and chemical structure of target cells [21,22,23]. However, apart from these significant properties, the major limitation preventing the use of TiO_2_NPs is the formation of milky suspensions in water, which need expensive liquid–solid separation [24]. Therefore, using TiO_2_NPs as antibacterial agents requires the assistance of other biomaterials as supports. There are many examples of scaffold materials, such as silica, polymeric materials, glass and activated carbon, used for delivering TiO_2_NPs [25,26]. Among them, hydrogels are the most novel scaffolds for TiO_2_NPs delivery [27]. 

Hydrogels consist of three-dimensional networks that are able to absorb and retain liquids while maintaining their shape, which is a paramount feature in biological applications and antibacterial dressings [28,29,30]. Self-assembling peptide hydrogels are a class of hydrogels that are extremely attractive for biomedical applications due to their biocompatibility, biodegradability and ability to give rise to biomimetic structures (i.e., capable of mimicking some properties of extracellular matrices). In addition, their formation is based on the spontaneous organization of peptides as building blocks, forming stable networks [31,32]. The use of self-assembling peptides as building blocks of structures such as hydrogels brings many advantages, including fast chemical synthesis, adaptation to different environmental conditions (i.e., pH, temperature and ionic strength), well-known structure and function of different peptides and proteins and the availability of secondary structures (helical or *β*-hairpin/sheet) that facilitate self-assembly [33]. Furthermore, self-assembly eliminates the use of toxic cross-linking agents during gel preparation. Peptide hydrogels are currently used in various fields of regenerative medicine, especially tissue engineering, due to their ability to entrap water, which makes them, in some ways, very similar to biological tissues [34], allowing the preparation of scaffolds with specific physical and chemical properties based on the type of application required. Furthermore, peptide hydrogels can be used as carriers in drug and gene delivery since, given their porous structure, they allow the entrapment of different molecules (such as drugs, nucleic acids and metal nanoparticles) with antibacterial activity [35,36,37]. 

Previous studies unveiled the potential of TiO_2_NPs for the photocatalytic inactivation of microorganisms such as *Escherichia coli*, *Lactobacillus acidophilus* and *Staphylococcus aureus* [38]. Such studies have prompted the exploration of TiO_2_NPs’ disinfection efficacy on various microbial strains. There is a growing emphasis on the implications of embedding TiO_2_NPs within surfaces. Some researchers suggest that UV-light-exposed surfaces coated with TiO_2_NPs could redefine disinfection methods. This photooxidation technique via TiO_2_ emerges as a novel microbial removal approach, especially in domains where traditional biocides fall short or face restrictions [39]. However, TiO_2_NPs’ bactericidal potency is heightened in suspension compared to when integrated into surfaces. The reason is ostensibly the enhanced contact with microbial cells in suspension, which amplifies ROS generation, a factor underscored in numerous studies [38]. Furthermore, HeLa cells exposed to minimal concentrations of TiO_2_NPs exhibit an elevated risk of bacterial infiltration [40]. HeLa cells treated with specific TiO_2_NPs demonstrated a significant uptake of *S. aureus* per cell, posing further infection threats. This increase was linked to TiO_2_NPs absorbing bacterial polysaccharides and a spike in extracellular LDH. Conversely, TiO_2_NP-exposed macrophages consumed significantly fewer bacteria, amplifying infection risks [40].

This study developed and characterized a novel hydrogel composite based on a self-assembling tripeptide (Fmoc-Phe_3_) containing TiO_2_NPs (hgel-TiO_2_NPs). To the best of our knowledge, this is the first report in which a self-assembling peptide hydrogel is used as a scaffold for stabilizing TiO_2_NPs. According to our previous studies, the antibacterial activity of Fmoc-Phe_3_ hydrogel is negligible, but the addition of TiO_2_NPs may increase the antimicrobial properties of the composite. In the present work, different concentrations of TiO_2_NPs inside the hydrogels were used in order to investigate the effect of concentration on the swelling ability, mechanical strength and antibacterial properties of the composites. In addition, the effect of ultrasonication during hydrogel preparation on the properties of the composites was evaluated.

## 2. Results and Discussion

### 2.1. Preparation of Hgel-TiO_2_ Composites

**Figure 1:**
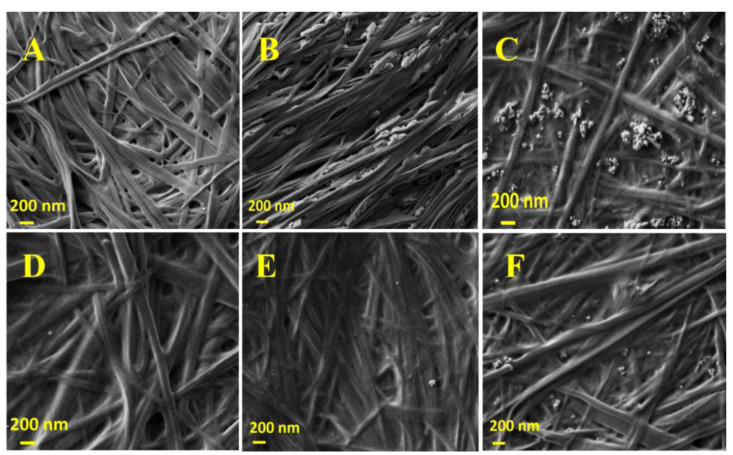
SEM images of: (**A**) hgel, (**B**) hgel-TiO_2_NPs(5 mg), (**C**) hgel-TiO_2_NPs(10 mg) in the absence of sonication and (**D**) hgel, (**E**) hgel-TiO_2_NPs(5 mg), (**F**) hgel-TiO_2_NPs(10 mg) with sonication.

Fmoc-Phe_3_ self-assembling peptide and TiO_2_NPs were chosen to prepare hgel-TiO_2_NP composites. the biosynthesis of Fmoc-Ph_3_ hydrogel using Fmoc-Phe and Phe_2_ as starting reagents and a lipolytic enzyme (PFL) to catalyze the formation of a peptide bond between them was optimized in previous studies [41]. In the present work, this reaction was used to prepare hydrogels and impregnate them with different amounts of TiO_2_NPs. Ultrasonication was also applied to the samples and its effect on several physicochemical properties of the obtained hydrogels was investigated. It was observed that all hydrogel samples were formed after 30 min of incubation at 30 °C. Field emission scanning electron microscopy (FESEM) studies were conducted to investigate the effect of sonication on the hydrogel fibrillary structures, as well as the dispersion of different amounts of TiO_2_NPs inside the hydrogels. Figure 1A–C show the fibrillar structure of hgel-TiO_2_NPs (5 mg) and hgel-TiO_2_NPs (10 mg) before applying sonication, respectively. As evident, TiO_2_NPs showed aggregation in both concentrations inside the gel. At the same time, after sonication (Figure 1E,F), they were finely dispersed without any aggregation, which reveals the effect of sonication on NP dispersion. It is also interesting to mention that sonication did not affect the fibrillar structure of hgel (Figure 1D) and hgel-TiO_2_NPs. 

### 2.2. Dynamic Light Scattering (DLS)

**Figure 2:**
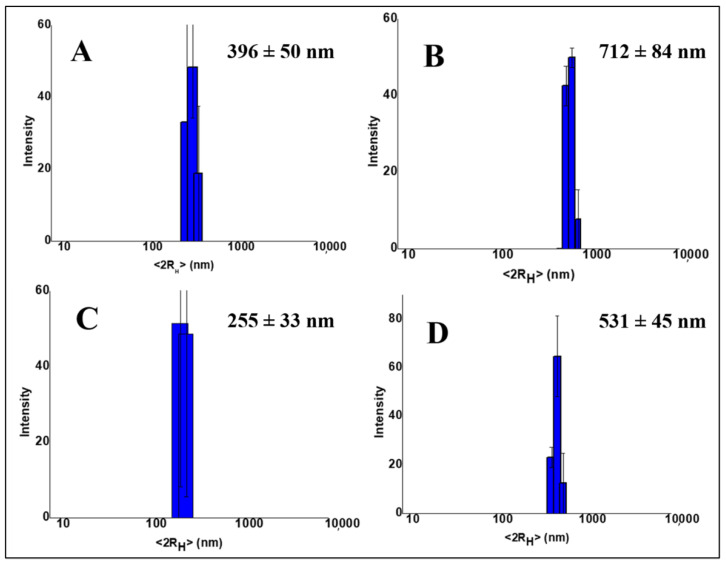
Size distributions of (**A**) hgel-TiO_2_(5 mg); (**B**) hgel-TiO_2_(10 mg); (**C**) hgel-TiO_2_(5 mg)-s; (**D**) hgel-TiO_2_(10 mg)-s (“s” represents sonication).

DLS measurements were performed to evaluate the size and polydispersity of TiO_2_NPs once they were inserted inside the hydrogels and evaluate if they underwent aggregation. Figure 2A,B show the size distribution of hgel-TiO_2_NP composites containing different amounts of TiO_2_NPs (5 mg and 10 mg, respectively), prepared without sonication. The average diameters measured for these samples were 396 ± 50 nm and 712 ± 84 nm, respectively. However, after sonication (Figure 2C,D) the particle size decreased (255 ± 33 nm and 531 ± 45 nm, respectively), demonstrating the positive effect of sonication on the dispersion of particles, which is consistent with SEM data. 

### 2.3. X-ray Small-Angle Scattering (SAXS)

SAXS profiles were collected to study the structural features within the 1–100 nm scale of hgel and hgel-TiO_2_NPs, both sonicated and non-sonicated and with different concentrations of TiO_2_NPs. We can mention that the visual appearance of the samples was slightly inhomogeneous at a macroscopic (mm) scale, showing regions with a more intense white color probably related to a higher local concentration of TiO_2_NPs; for SAXS data collection, we sampled 0.5 mm portions and therefore slight variations of signal can be considered to be due to this variability. The data of the pristine hydrogel (hgel, grey and black dots) show the profile of fibrillar structures, with a characteristic cross-section radius of the cylinder-like fibrillar aggregates in the range of 2.5–8 nm. For q < 0.2 nm^−1^, the slope deviates from the q^−1^ predicted for straight rod-like objects towards more negative values (close to −2) since the fibrils cannot be seen as individual rods anymore at larger length scales (>10–20 nm) compared to their cross-sections. This could be interpreted as being due to the branched fibrillar network, giving rise to a fractal structure with a characteristic dimension close to 2.

**Figure 3:**
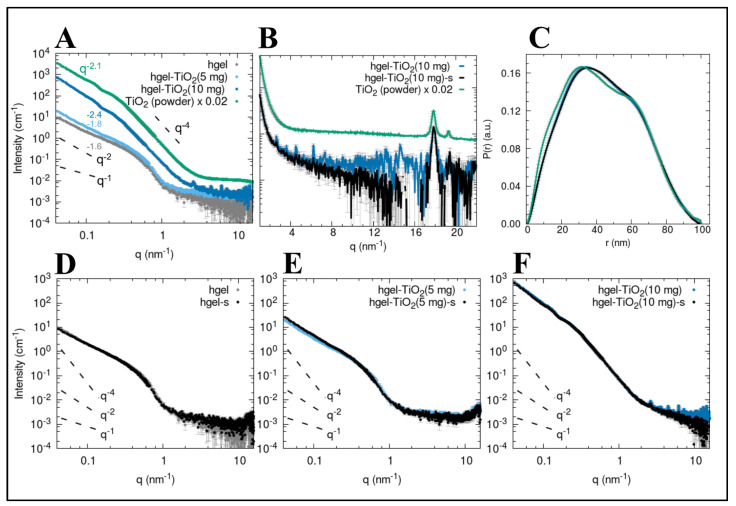
SAXS profiles of (**A**) hgel (grey dots), hgel-TiO_2_(5 mg) (light blue dots), hgel-TiO_2_(10 mg) (blue dots) and of the TiO_2_ powder (green dots). Characteristic power laws are reported for reference (black dashed lines), while the exponent observed at q < 0.3 nm^−1^ is reported in each case. (**B**) Enlargement of the WAXS region of the scattering profile to highlight diffraction peaks for the samples hgel-TiO_2_(10 mg) (blue), hgel-TiO_2_(10 mg)-s (black) and TiO_2_ powder (green). (**C**) Pair distance distribution functions obtained from the SAXS data of the samples hgel-TiO_2_(10 mg) (blue), hgel-TiO_2_(10 mg)-s (black) and of the TiO_2_ powder (green). Scaling factors were applied to match the height of the main maximum: 1.125 for hgel-TiO_2_(10 mg)-s and 0.0044 for TiO_2_ powder. (**D**–**F**) Comparison between the SAXS profiles of non-sonicated and sonicated samples.

As can be seen in Figure 3A, the SAXS profile obtained for the hgel-TiO_2_NPs at low concentration is almost superimposable to that of the hydrogel sample in the q-range > 0.3 nm^−1^, suggesting that the fibrillar cross-section is not perturbed. A slightly higher scattering intensity can be appreciated for q < 0.3 nm^−1^, with initial power law q^−1.77^, compared to q^−1.63^ observed for the pristine hgel obtained in the same conditions. When considering the composite sample with double the content of TiO_2_ particles (hgel-TiO_2_NPs (10 mg)), a very intense additional scattering signal with initial slope oscillations (maxima at q 0.125, 0.22 and 0.3 nm^−1^) can be seen in the lower q. A comparison with the SAXS profile of the TiO_2_NP commercial powder clarifies that for the high concentration composite most of the signal can be attributed to the inorganic particles, which have an intrinsically much higher scattering power compared to the peptide hgel. The relative amount of this contribution seems not to be linearly related to the nominal volume fraction of TiO_2_ added in the sample, since the scale factor for roughly reproducing the SAXS signal of the low-concentration composite as a sum of the signals from hgel and TiO_2_ particles should be only 0.04 times that of the high-concentration composite, while the TiO_2_ amount should be only half. Meanwhile, at q > 0.3 nm^−1^ the SAXS data of the TiO_2_NP powder follows Porod’s power law decay (q^−4^), indicating that the individual particles have a sharp interface when seen at a length scale < 10–20 nm, and the initial power law is close to q^−2.1^. This suggests that the individual TiO_2_ particles are clustered in mass fractals with a fractal dimension of 2.1. The pair distance distribution functions P(r) (Figure 3C) can help estimate the characteristic sizes that contribute to the SAXS profile of the TiO_2_NPs, even if the overall size of the inorganic nanoparticle clusters is beyond the largest accessible distance of the SAXS experiment (>π/0.04 nm^−1^ ≈ 80 nm), as also perceived from the SEM micrographs. The P(r) functions present a main maximum around 30 nm, which is coherent with the approximate radius expected for the nanoparticles, but also contributions of distances around 12 nm and 60 nm can be seen. This might be due to two additional populations of particles and to a hierarchical structuring of the clusters made by particles with approximate diameters of 25 and 60 nm organized in a non-compact aggregate. Diffraction peaks can also be observed in the scattering profiles of the composites (Figure 3B). While the intense peak at 17.8 nm^−1^ and the following one at 19.3 nm^−1^ are also found in the scattering pattern of the TiO_2_NP powder and are consistent with those expected for the anatase crystalline phase [42], additional peaks are observed for q < 17.8 nm^−1^ in the composites with the highest TiO_2_ content. These signals (peaks at q values 2.65, 3.63, 4.53, 6.21, 7.32, 14.64 nm^−1^) suggest the presence of mesoscale ordered structures (lamellar, hexagonal phases) with characteristic spacings of the order of 2 nm, which cannot be found in dry TiO_2_ powder but form in the composite hydrogel preparations.

As can be seen in Figure 3D,E, the data of both the pristine hydrogel and of the hgel-TiO_2_NPs in both concentrations show overall the same profile when comparing non-sonicated vs. sonicated samples, indicating that sonication does not have any effect on the hydrogel structure nor appreciable effects on the composite at a low concentration of TiO_2_. For the high-concentration hgel-TiO_2_NPs, the sonicated sample shows less intense peaks in the q region of 2–10 nm^−1^, suggesting that the sonication procedure might affect the amount of the ordered mesophases formed.

### 2.4. Rheological Studies

**Figure 4:**
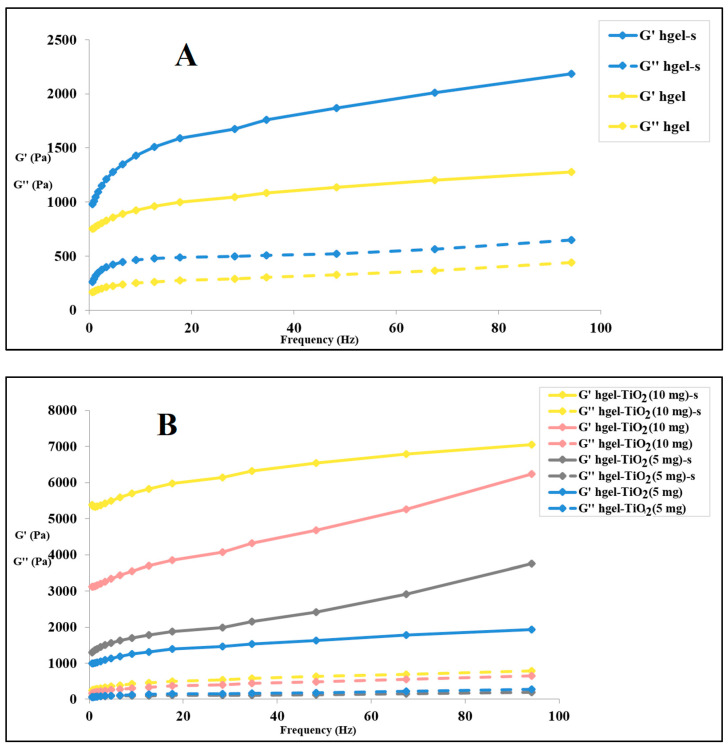
Frequency sweep of (**A**) hydrogel prepared with or without sonication, (**B**) hgel-TiO_2_NPs with different concentrations of NPs prepared with or without sonication (“s” in the insets indicates sonication).

This investigation aimed to unravel the effect of TiO_2_NPs on the viscoelastic properties of the hydrogels, which are essential for their final application. As can be seen in Figure 4A,B, all sonicated samples show higher G′ (elastic modulus) and G″ (viscous modulus) values in comparison to their non-sonicated counterparts (the only exception is for hgel-TiO_2_NPs (5 mg), where G″ overlaps in both sonicated and non-sonicated samples) which show the effect of sonication in providing homogenized matrices and fibrillar structures that may increase the tensile strength of the hydrogels both in the presence and absence of NPs. Regarding Figure 4B, when increasing the concentration of TiO_2_NPs inside the hydrogel, the G′ value rises, demonstrating the formation of further cross-links leading to a stronger hydrogel. 

### 2.5. Swelling Ability 

**Figure 5:**
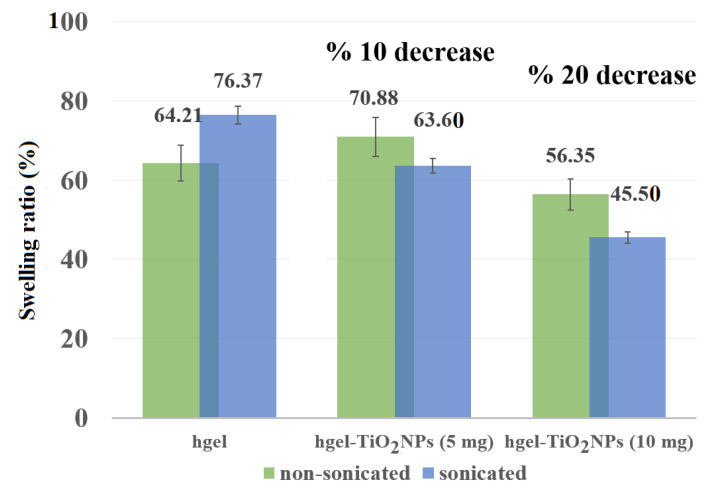
Swelling ratio of hgel, hgel-TiO_2_NPs (5 mg) and hgel-TiO_2_NPs (10 mg), prepared with or without sonication.

The swelling behaviors of the native hydrogel and hgel-TiO_2_NPs were investigated. Different samples were prepared, with or without sonication, to determine the effect of this treatment on the swelling ability of the obtained hydrogel materials. As shown in Figure 5, the sonicated hydrogel showed a higher swelling degree than its non-sonicated counterpart, while for the hydrogel composites non-sonicated samples had the highest swelling degree. Overall, by increasing the concentration of TiO_2_NPs, the swelling ability decreased (as we saw in Section 2.4, the tensile strength increased in this condition), which may indicate that the presence of NPs provides cross-links and prevents water molecules from easily penetrating inside the matrix. For the case of sonicated samples, they showed even lower swelling degrees in comparison to non-sonicated ones. Probably, since sonication leads to a higher dispersity of TiO_2_NPs and a more homogeneous hydrogel matrix, such uniform hydrogel networks show higher tensile strength (Section 2.4) and consequently a lower swelling degree than non-sonicated ones. For hgel-TiO_2_NPs (5 mg), there was an almost 10% decrease in swelling ability after sonication that doubled for hgel-TiO_2_NPs (10 mg).

### 2.6. Antibacterial Studies

**Figure 6:**
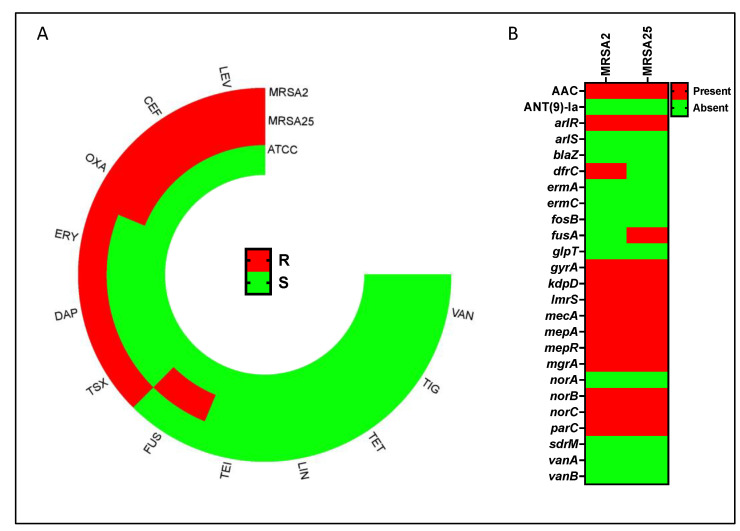
Antimicrobial resistance genes and antimicrobial susceptibility test. (**A**) Circular heatmap of antimicrobial susceptibility testing (AST) for the following antibiotics: ceftaroline (CEF), daptomycin (DAP), erythromycin (ERY), fusidic acid (FUS), levofloxacin (LEV), linezolid (LIN), oxacillin (OXA), teicoplanin (TEI), tetracycline (TET), tigecycline (TIG), trimethoprim–sulfamethoxazole (TSX) and vancomycin (VAN), expressed as resistant (red—R) or susceptible (green—S). (**B**) Heatmap displaying antibiotic resistance genes found in the methicillin-resistant *S. aureus* (MRSA) strains (MRSA2 and MRSA25) and the laboratory *S. aureus* strain ATCC 6538 (ATCC).

MRSA represents a major antibiotic-resistant pathogen involved in tissue and medical-device-related infections [43]. TiO_2_NPs have shown potential antimicrobial properties [44], so their antibacterial effect was tested on MRSA clinical isolates. To initially characterize the virulence of the bacterial isolates, antimicrobial susceptibility testing (AST) was performed for ceftaroline (CEF), daptomycin (DAP), erythromycin (ERY), fusidic acid (FUS), levofloxacin (LEV), linezolid (LIN), oxacillin (OXA), rifampicin (RIF), teicoplanin (TEI), tetracycline (TET), tigecycline (TIG), trimethoprim–sulfamethoxazole (TSX), and vancomycin (VAN). Notably, MRSA2 and MRSA25 showed resistance to 50% (6/12) and 33.3% (4/12) of the tested antibiotics, respectively. Conversely, the laboratory strain *S. aureus* 6583 (ATCC) was susceptible to all antibiotics (Figure 6A). Antibiotic resistance genes were assessed for all clinical isolates (Figure 6B). The MRSA phenotype was confirmed by the presence of the *mecA* genes conferring resistance to oxacillin or ceftaroline. Resistance to macrolides and lincosamides was associated with erm(C) or erm(A). In MRSA2 and MRSA25, resistance to LEV was linked to the *gyrA* gene. Resistance to TSX and fusidic acid was associated with the *dfrC* (MRSA2) and fusA (MRSA25) genes, respectively (Figure 6A).

**Figure 7:**
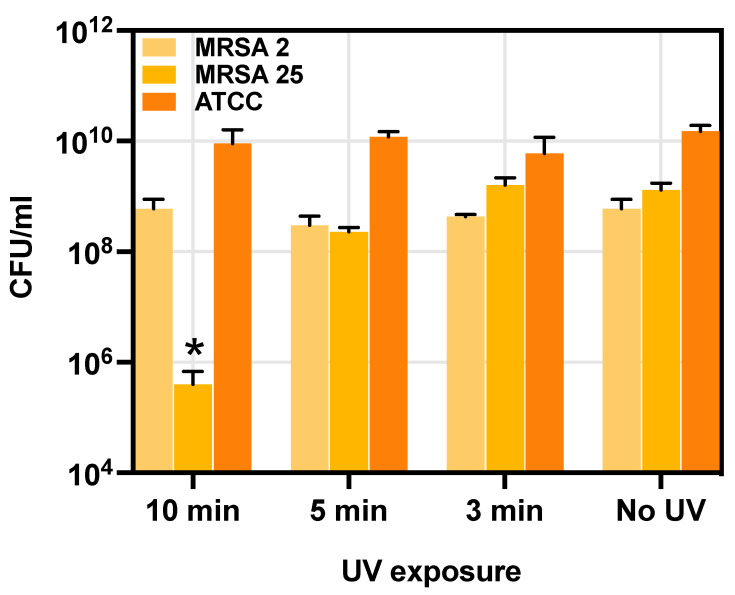
Effect of UV light at different exposure times on the growth curve of *S. aureus*. The graph represents 10 min, 5 min and 3 min UV exposure compared to unexposed cells. Each value corresponds to the mean of 3 replicates. The error bars represent standard deviation. Statistical differences were determined using the Kruskal–Wallis test. *, *p* < 0.05.

A preliminary study determined the UV exposure time that would not impact bacterial growth. *S. aureus* suspensions in nutrient broth were exposed to UV light at an intensity of 6.9 mW/cm^2^ for 10, 5 and 3 min. Notably, a 10 min exposure significantly reduced the growth of the MRSA25 strain (*p* = 0.037) compared to untreated controls. In contrast, 5 and 3 min exposures did not impact *S. aureus* growth (Figure 7). Based on these results, a 5 min UV exposure was selected as the optimal treatment for the photoactivation of TiO_2_NPs. 

**Figure 8:**
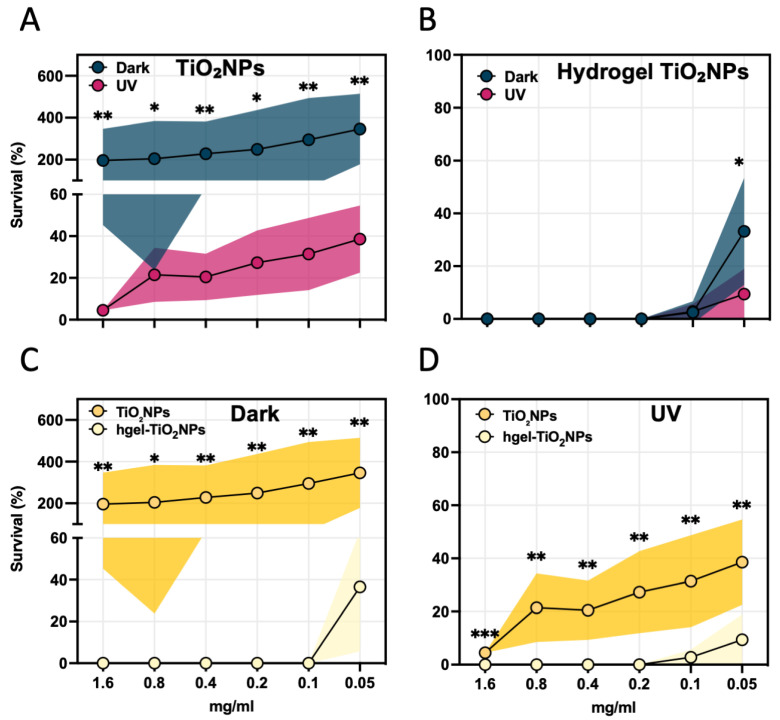
Antibacterial activity of photoactivated (UV light) and non-photoactivated (dark) (**A**) TiO_2_NPs and (**B**) hgel-TiO_2_NPs at different concentrations of TiO_2_ (0.05, 0.1, 0.2, 0.4, 0.8 and 1.6 mg/mL). Comparative analysis of antibacterial activity for TiO_2_NPs and hgel-TiO_2_NPs in the dark (**C**) or in the presence of UV (**D**). Values are expressed as a percentage of survival of treated cells compared to the untreated controls. Data points and the corresponding shaded areas represent the mean ± standard deviation from three independent experiments, each analyzed in triplicate. Statistical differences were determined using the Mann–Whitney test. *, *p* < 0.05; **, *p* < 0.01.

The inhibitory effects of different concentrations (0.05, 0.1, 0.2, 0.4, 0.8 and 1.6 mg/mL) of TiO_2_NPs and hgel-TiO_2_NPs on *S. aureus* strains are shown in Figure 8. Specifically, after UV activation, *S. aureus* strains exposed to TiO_2_NPs showed a more significant reduction in surviving cells than those in dark conditions across all tested concentrations (Figure 8A). Notably, bacterial cells exposed to TiO_2_NPs in dark conditions showed an unexpected increase in CFU/mL compared to the untreated control cells (Figure 8A). In contrast, *S. aureus* bacterial cultures exhibited a marked concentration-dependent survival decrease in both photoactivated and non-photoactivated hgel-TiO_2_NPs (Figure 8B). It is worth noting that at the lower tested concentration (0.05 mg/mL), photoactivated hgel-TiO_2_NPs resulted in a significant (*p* = 0.026) reduction in CFU/mL compared to dark conditions.

Comparative analysis revealed that hgel-TiO_2_NPs significantly reduced *S. aureus* survival compared to TiO_2_NPs, both in the dark (Figure 8C) and photoactivated (Figure 8D) conditions, across all tested concentrations.

**Figure 9:**
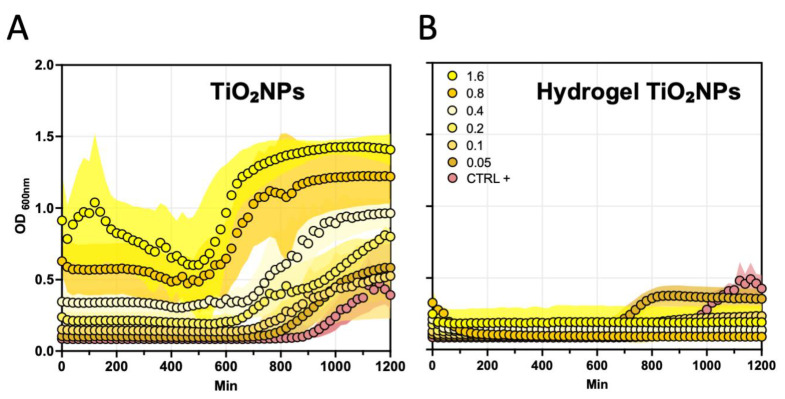
Metabolic activity of *S. aureus* strains in response to varying concentrations of TiO_2_NPs (0.05, 0.1, 0.2, 0.4, 0.8 and 1.6 mg/mL) using both non-photoactivated (**A**) TiO_2_NPs and (**B**) hgel-TiO_2_NPs, assessed by resazurin-to-resorufin conversion after 1200 min of incubation. Data points and the corresponding shaded areas represent the mean ± standard deviation. Data represent the means ± standard errors from two independent experiments, each analyzed in duplicate.

The impact of non-photoactivated TiO_2_NPs and hgel-TiO_2_NPs on the metabolic activity of *S. aureus* was evaluated at different TiO_2_NPs concentrations (0.05, 0.1, 0.2, 0.4, 0.8 and 1.6 mg/mL). This was determined by monitoring the transformation of resazurin to resorufin over a 1200 min period, as depicted in Figure 9. When exposed to non-photoactivated TiO_2_NPs, *S. aureus* isolates exhibited a concentration-dependent increase in their metabolic activity (Figure 9A). This result supports the previous observations of increased bacterial growth in the presence of non-photoactivated TiO_2_NPs. Conversely, the response to non-photoactivated hgel-TiO_2_NPs remained consistently low across most concentrations, except for 0.05 mg/mL (Figure 9B). This finding aligns with a plate count assay that indicated partial bacterial growth at the hgel-TiO_2_NP concentration of 0.05 mg/mL.

The obtained results underpin the importance of understanding environmental influences when considering therapeutic NP deployment. The observed influence of TiO_2_NPs on *S. aureus* growth, specifically the unexpected rise in bacterial growth in non-photoactivated TiO_2_NP conditions, resonates with findings from a previous study where HeLa cells exposed to low concentrations of TiO_2_NPs exhibited heightened sensitivity to *S. aureus* infections [40,45]. This alignment with previous studies underscores a potentially complex interplay between TiO_2_NPs and bacterial growth dynamics, dependent on the specific conditions and cellular context. Adding to this concern is the widespread use of TiO_2_NPs in cosmetics that has raised safety concerns [46]. 

The efficacy of hgel-TiO_2_NPs in diminishing *S. aureus* populations, regardless of photoactivation, is another crucial observation. This concentration-driven effect demonstrates that hgel-TiO_2_NPs have intrinsic antistaphylococcal properties that can act independently of light exposure, a finding consistent with the broader understanding of NPs’ interactions with bacteria [44,47]. Furthermore, when combined with photoactivation, hgel-TiO_2_NPs displayed augmented antibacterial activity, even at suboptimal concentrations like 0.05 mg/mL. This interplay between NP concentration and UV light exposure has been explored previously, reinforcing the role of UV light in enhancing NP-based antimicrobial strategies [48]. The present study also offers insightful revelations into how non-photoactivated TiO_2_NPs and hgel-TiO_2_NPs distinctly influence the metabolic activity of *S. aureus*. The concentration-dependent growth associated with TiO_2_NPs suggests that non-photoactivated TiO_2_NPs might enhance bacterial metabolic activity. While supported by the data, this assertion necessitates further investigations to ascertain the underlying mechanisms. 

## 3. Conclusions

In this study, we synthesized a novel composite scaffold containing TiO_2_NPs that exhibited increased antibacterial properties. The results of SEM demonstrated that the tripeptide hydrogel was able to stabilize TiO_2_NPs. We also applied sonication to the composites before gel formation and, based on SEM and SAXS results, sonication did not affect the hydrogel fibrillary structure but a higher dispersion of TiO_2_NPs was observed. Rheology experiments showed that by increasing the concentration of TiO_2_NPs the mechanical strength of the hydrogels increased. In addition, sonication positively affected tensile strength, while the results of swelling ability followed the reverse trend. TiO_2_NPs showed poor antimicrobial activity in dark conditions independently from their concentration. A pivotal observation was the unanticipated increase in bacterial growth with TiO_2_NPs in dark conditions, highlighting potential risks associated with NP application. Hgel-TiO_2_NPs consistently inhibited bacterial growth, with or without UV light activation, showing significantly higher efficacy than TiO_2_NPs. The discernible difference in antimicrobial potency between these NP formulations provides an avenue for research targeting improved antimicrobial agents, especially in the light of rising antibiotic resistance.

## 4. Materials and Methods

### 4.1. Materials 

*N*-(9-Fluorenylmethoxycarbonyl)-phenylalanine (Fmoc-Phe, 99%, 387.44 g/mol) and diphenylalanine (Phe_2_, 98%, 312.36 g/mol) were purchased from Bachem GmbH (Weil am Rhein, Germany) and used as received. Lipase from Pseudomonas fluorescens (PFL ≥ 20,000 U/mg), titanium (IV) oxide anatase nanopowder (TiO_2_NPs, 99.7%, diameter < 25 nm) and all other chemicals and solvents were obtained from Sigma Aldrich (St. Louis, MO, USA) and used as received.

### 4.2. Preparation of Hydrogel Composites

FmocPhe_3_ hydrogel (hgel) preparation was carried out according to previous works [30]. To prepare the hydrogel composites containing TiO_2_NPs (hgel-TiO_2_NPs), the reagents used for hydrogel formation, Fmoc-Phe and Phe_2_, were added to a suspension of TiO_2_NPs. Two TiO_2_NP concentrations were used: 1.67 mg/mL and 3.33 mg/mL. After adding the reagents, the pH of the suspension was adjusted to ≈9 by adding 0.5 M NaOH. The suspension was stirred magnetically for 10 min and then neutralized until reaching pH ≈ 7 using 0.1 M HCl. The final volume of the mixture was adjusted to 3 mL. Finally, 100 µL of 50 mg/mL PFL solution was added, followed by a 30 min incubation in a thermostatic bath at 30 °C, which allowed hydrogel formation. Ultrasonic treatment was applied to the samples before the neutralization step (10 min, 40 KHz), placing the suspensions in an ultrasonic bath (Ultrasonic Cleaner, DU-06, 50 W, ArgoLab, Carpi (MO), Italy) before proceeding as described above. 

### 4.3. Field Emission Scanning Electron Microscopy (FESEM) Measurements

FESEM images were acquired in the CNIS laboratory of Sapienza University using a variable pressure SEM (VP-SEM, SU-3500, Hitachi, Chiyoda, Japan). Samples were deposited on silicon stubs, dried at room temperature and analyzed at low accelerating voltage to avoid radiation damage of the samples. 

### 4.4. Dynamic Light Scattering (DLS)

DLS measurements were performed using a Zetasizer Nano S (Malvern Instruments, Malvern, UK) with a 4 mW He-Ne laser (633 nm). Hydrogel samples were gently broken and 50 µL of each sample was mixed with a fixed amount of distilled water to provide transparent and dilute suspensions. All measurements were performed three times with at least 10 replications at 25 °C. To calculate the average hydrodynamic diameter, peak intensity analysis was used. 

### 4.5. Small-Angle X-ray Scattering (SAXS)

SAXS measurements were carried out based on previously published works with some modifications [49]. A Xeuss 2.0 Q Xoom instrument (Xenocs SA, Grenoble, France) was used. A small portion of each hydrogel sample was inserted into borosilicate glass capillaries and the measurements were carried out at 25 ± 1 °C and reduced pressure (<0.4 mbar). Model intensities were calculated using SasView [50]. In order to obtain information on the size in the real space of the inhomogeneities giving rise to SAXS profiles, pair distance distribution functions were obtained by indirect Fourier inversion of the I(q) using the GNOM software of the ATSAS package [51]. 

### 4.6. Rheological Measurements

A rotational rheometer (MCR 302, Anton Paar, Graz, Austria) with a plate–plate geometry was used to record elastic (G′) and viscous (G″) moduli of hydrogel samples. The experiments were carried out in frequency sweep, subjecting the sample to deformation with constant intensity (1%) and varying the oscillation frequency. Measurements were performed at constant temperature (30 °C) and maintaining the plates at a 1 mm distance.

### 4.7. Swelling Ability

Swelling degree studies of the hydrogels were performed by adding 3 mL of PBS (pH = 7.4) to each hydrogel and incubating at 37 °C. After 24 h, PBS was removed and each hydrogel sample was weighed (W_s_). Then, the hydrogels were freeze-dried and weighed again (W_d_). The swelling ratios (q) were calculated using the following equation:

(1) $$q=(W_{s}-W_{d})/W_{d}$$

### 4.8. Tested Microorganisms

*S. aureus* ATCC 6538 was purchased from the American Type Culture Collection. (Manassas, VA, USA) Methicillin-resistant *Staphylococcus aureus* (MRSA) clinical isolates were provided by the Microbial Strain Repository of the Microbiology and Virology Laboratory of San Gallicano Dermatological Institute IRCCS, Rome, Italy. These microorganisms were collected in 2020 from patients presenting skin and soft tissue infections. MRSA strains presented the gene for methicillin resistance (mecA), oxacillin resistance (MIC ≥ 4 mg/mL) and a positive agglutination test for penicillin-binding protein (PBP2; Oxoid, Basingstoke, UK). Whole-genome analysis of MRSA strains and the phylogenomics were carried out as previously described [52]. 

### 4.9. Determination of Minimal Inhibitory Concentration (MIC)

The antimicrobial activity of hgel-TiO_2_NPs and TiO_2_NPs was determined for each strain using the broth microdilution method to define the minimum inhibitory concentration (MIC). Briefly, a standard bacterial inoculum of approximately 1 × 10^5^ CFU/mL was prepared in cation-adjusted Mueller–Hinton broth (MHB, Thermo Fisher Scientific, Waltham, MA, USA) and used to inoculate a 96-well polystyrene flat-bottom plate with 100 µL. Cells were treated with serial 2-fold dilutions of the compounds in MHB, ranging from 1.6 to 0.05 mg/mL. Growth controls containing no compounds and sterility controls without bacteria were also included.

To evaluate the bactericidal activity of UV light-activated hgel-TiO_2_NPs and TiO_2_NPs, a preliminary set of experiments was conducted using microplates exposed to direct UV light for 10, 5 and 3 min [38]. The plates were incubated for 24 h at 37 °C in a microplate reader (Multiskan SkyHigh, Thermo Fisher Scientific, Waltham, MA, USA), where QD600 readings were taken every 20 min. Viable cell counts were determined through plate counting to measure CFU/mL. Experiments were performed in triplicate and repeated three times.

### 4.10. Determination of Metabolic Activity

To determine the metabolic activity, 100 µL of a standard bacterial inoculum of approximately 1 × 10^5^ CFU/mL bacteria, treated with a two-fold dilution of the tested compounds as previously described (from 16 to 0.05 mg/mL), was placed in 96-well flat-bottom microtiter plates with the addition of resazurin (Promega, Madison, WI, USA). Positive controls of the bacterial inoculum were left untreated. Metabolic activity was assessed in the absence of UV exposure. The plates were incubated for 1200 min (20 h) at 37 °C and the absorbance (600 nm) was recorded every 20 min by a multidetector microplate reader (Multiskan SkyHigh, Thermo Fisher Scientific, Waltham, MA, USA) [53]. 

## Data Availability

Data are contained within the article.
